# Supplementary material for: Deletion of wfs1 Impairs Oligodendrocyte Precursor Cells Dorsal Distribution and Myelination Through the wfs1-hmgcs1 Axis in Zebrafish
Source: Biology (Basel). 2026 Aug 16;15(16):1402. doi: 10.3390/biology15161402 (PMC13510151; doi:10.3390/biology15161402)
Supplement: Supplementary file 1 [file biology-15-01402-s001.zip › File S1-Transcriptome sequencing data and differentially expressed genes (DEGs) in wfs1-- zebrafish at 5 dpf/2.KEGG/wfs1KOvsWT/GSEA/wfs1KO/PROPANOATE_METABOLISM/PROPANOATE_METABOLISM(DRE00640).html]

Details for gene set PROPANOATE\_METABOLISM(DRE00640)[GSEA]

|  || Dataset | deg.sample |
| Phenotype | sample.cls |
| Upregulated in class | wfs1KO |
| GeneSet | PROPANOATE\_METABOLISM(DRE00640) |
| Enrichment Score (ES) | 0.44995585 |
| Normalized Enrichment Score (NES) | 1.4988562 |
| Nominal p-value | 0.0 |
| FDR q-value | 0.21024777 |
| FWER p-Value | 0.637 |
Table: GSEA Results Summary

  

Fig 1: Enrichment plot: PROPANOATE\_METABOLISM(DRE00640)      
 Profile of the Running ES Score & Positions of GeneSet Members on the Rank Ordered List

  

| PROBE | DESCRIPTION (from dataset) | GENE SYMBOL | GENE\_TITLE | RANK IN GENE LIST | RANK METRIC SCORE | RUNNING ES | CORE ENRICHMENT || 1 | 437019\_1 | pcca |  |  | 307 | 3.602 | 0.1373 | Yes |
| 2 | 317746 | suclg2 |  |  | 871 | 2.285 | 0.2129 | Yes |
| 3 | 553401 | hadhaa |  |  | 1608 | 1.593 | 0.2548 | Yes |
| 4 | 559403\_1 | acaca |  |  | 1982 | 1.374 | 0.2991 | Yes |
| 5 | 103909985 | LOC103909985 |  |  | 3098 | 1.013 | 0.3054 | Yes |
| 6 | 436914 | acss2 |  |  | 3398 | 0.954 | 0.3350 | Yes |
| 7 | 553804 | mcee |  |  | 3424 | 0.947 | 0.3728 | Yes |
| 8 | 445288 | acads |  |  | 3897 | 0.848 | 0.3926 | Yes |
| 9 | 100000859 | ehhadh |  |  | 4565 | 0.737 | 0.4017 | Yes |
| 10 | 554124 | bckdha |  |  | 5080 | 0.670 | 0.4129 | Yes |
| 11 | 559403 | acaca |  |  | 5108 | 0.666 | 0.4392 | Yes |
| 12 | 317746\_1 | suclg2 |  |  | 5836 | 0.604 | 0.4411 | Yes |
| 13 | 449662 | acox1 |  |  | 6280 | 0.559 | 0.4500 | Yes |
| 14 | 436747 | ldhbb |  |  | 6973 | 0.494 | 0.4484 | No |
| 15 | 566019 | acss3 |  |  | 7611 | 0.446 | 0.4466 | No |
| 16 | 436647 | aldh6a1 |  |  | 9402 | 0.340 | 0.4042 | No |
| 17 | 793834 | hadhab |  |  | 10077 | 0.298 | 0.3952 | No |
| 18 | 437019 | pcca |  |  | 12058 | 0.187 | 0.3407 | No |
| 19 | 406421 | acox3 |  |  | 12372 | 0.170 | 0.3378 | No |
| 20 | 541435\_1 | acss1 |  |  | 12397 | 0.169 | 0.3440 | No |
| 21 | 101882140 | LOC101882140 |  |  | 13313 | 0.125 | 0.3204 | No |
| 22 | 30497 | ldhba |  |  | 13522 | 0.114 | 0.3185 | No |
| 23 | 541388\_1 | dbt |  |  | 15004 | 0.046 | 0.2739 | No |
| 24 | 406299 | sucla2 |  |  | 15431 | 0.025 | 0.2615 | No |
| 25 | 569980 | bckdhb |  |  | 15788 | 0.007 | 0.2506 | No |
| 26 | 405861 | pccb |  |  | 17313 | -0.046 | 0.2047 | No |
| 27 | 554124\_1 | bckdha |  |  | 17916 | -0.072 | 0.1887 | No |
| 28 | 556236 | acacb |  |  | 18028 | -0.077 | 0.1884 | No |
| 29 | 368912 | echs1 |  |  | 18310 | -0.089 | 0.1832 | No |
| 30 | 378968 | abat |  |  | 19919 | -0.162 | 0.1393 | No |
| 31 | 436850 | suclg1 |  |  | 22189 | -0.278 | 0.0794 | No |
| 32 | 569581 | mut |  |  | 22328 | -0.286 | 0.0868 | No |
| 33 | 30496 | ldha |  |  | 22490 | -0.295 | 0.0938 | No |
| 34 | 541435 | acss1 |  |  | 22524 | -0.297 | 0.1048 | No |
| 35 | 399479 | dldh |  |  | 25027 | -0.448 | 0.0446 | No |
| 36 | 541388 | dbt |  |  | 25050 | -0.451 | 0.0623 | No |
| 37 | 560020 | acss2l |  |  | 26041 | -0.533 | 0.0529 | No |
| 38 | 798364 | hibch |  |  | 27149 | -0.642 | 0.0444 | No |
| 39 | 317746\_2 | suclg2 |  |  | 27320 | -0.651 | 0.0656 | No |
| 40 | 100037349 | mlycd |  |  | 28119 | -0.759 | 0.0715 | No |
| 41 | 553585 | echdc1 |  |  | 29752 | -1.149 | 0.0671 | No |
Table: GSEA details [plain text format]

  

Fig 2: PROPANOATE\_METABOLISM(DRE00640)      
 Blue-Pink O' Gram in the Space of the Analyzed GeneSet

  

Fig 3: PROPANOATE\_METABOLISM(DRE00640): Random ES distribution      
 Gene set null distribution of ES for **PROPANOATE\_METABOLISM(DRE00640)**
